# Perioperative Stroke in MCA Aneurysm Surgery: The Hidden Risks of Amphetamine Use

**DOI:** 10.3390/jcm14093246

**Published:** 2025-05-07

**Authors:** Firat Taskaya, Vanessa Magdalena Swiatek, Sifian Al-Hamid, Julius Reiser, Roland Schwab, Klaus-Peter Stein, Daniel Behme, Ali Rashidi, I. Erol Sandalcioglu, Belal Neyazi

**Affiliations:** 1 Department of Neurosurgery, Otto-von-Guericke University, 39120 Magdeburg, Saxony-Anhalt, Germany; firat.taskaya@st.ovgu.de (F.T.); vanessa.swiatek@med.ovgu.de (V.M.S.); sifian.al-hamid@st.ovgu.de (S.A.-H.); julius.reiser@st.ovgu.de (J.R.); klaus-peter.stein@med.ovgu.de (K.-P.S.); ali.rashidi@med.ovgu.de (A.R.); erol.sandalcioglu@med.ovgu.de (I.E.S.); 2 Department of Neuroradiology, Otto-von-Guericke University, 39120 Magdeburg, Saxony-Anhalt, Germany; roland.schwab@med.ovgu.de (R.S.); daniel.behme@med.ovgu.de (D.B.)

**Keywords:** amphetamine, perioperative stroke, middle cerebral artery aneurysm, substance abuse, vasospasm, neurosurgery

## Abstract

**Background/Objectives:** Perioperative strokes are a rare but recognized complication of cerebral aneurysm surgeries, often influenced by patient-specific factors. Amphetamine abuse, known for its vasospastic effects, is an underexplored risk factor in the neurosurgical setting. This report highlights the clinical and perioperative challenges associated with acute undisclosed amphetamine abuse in a patient undergoing elective clipping of an unruptured middle cerebral artery (MCA) aneurysm. **Methods:** A 46-year-old male presented with a 3 mm broad-based unruptured aneurysm in the proximal M1 segment of the right MCA. The patient reported a history of illicit drug use, including intravenous consumption. Upon further questioning, he admitted to intermittent use of amphetamines, although he denied any recent use. Elective aneurysm clipping via a transsylvian approach was performed after multidisciplinary consensus. Postoperatively, the patient developed anisocoria, prompting an emergency CT with perfusion and angiography, showing significant findings. Further imaging revealed a bilateral superior cerebellar artery territory infarction. Given the patient’s medical history, a toxicology screening later confirmed recent amphetamine use. **Conclusions:** This case highlights the need for preoperative evaluation, including routine toxicology screening, in patients with a history of substance abuse. Amphetamine use may present perioperative challenges and increase the risk of complications like vasospasm and stroke.

## 1. Introduction

Amphetamines are potent central nervous system stimulants with known cardiovascular and cerebrovascular effects, including vasospasm [1,2], hypertension, and an increased risk of stroke [3,4,5]. Chronic amphetamine use is associated with long-term vascular damage [6], while acute use can precipitate critical complications, particularly in high-stress medical situations such as surgery [7,8,9].

Despite discussions around potential worsening of the operative outcome caused by amphetamine use, it is concluded that discontinuing the amphetamine drug is not necessary [10]. Moreover, patients with a history of substance abuse often deny recent use, complicating perioperative risk assessments.

Here, we present the case of a 46-year-old man with a history of polysubstance abuse, including intravenous drugs such as heroin, which he had successfully discontinued. However, he admitted to ongoing amphetamine use and was explicitly advised to abstain from it in preparation for surgery. After a comprehensive preoperative evaluation of his substance use history and detailed anamnesis, the patient explicitly assured us he had ceased amphetamine consumption in preparation for surgery. However, subsequent investigations confirmed that he had consumed amphetamines both before and on the day of the surgical intervention. The patient was fully oriented and appeared credible preoperatively.

The patient presented for elective treatment of an unruptured proximal middle cerebral artery (MCA) aneurysm. Despite the instruction to cease amphetamine use, undetected use on the day of surgery contributed to a perioperative cerebellar stroke.

## 2. Case Report

**Figure 1:**
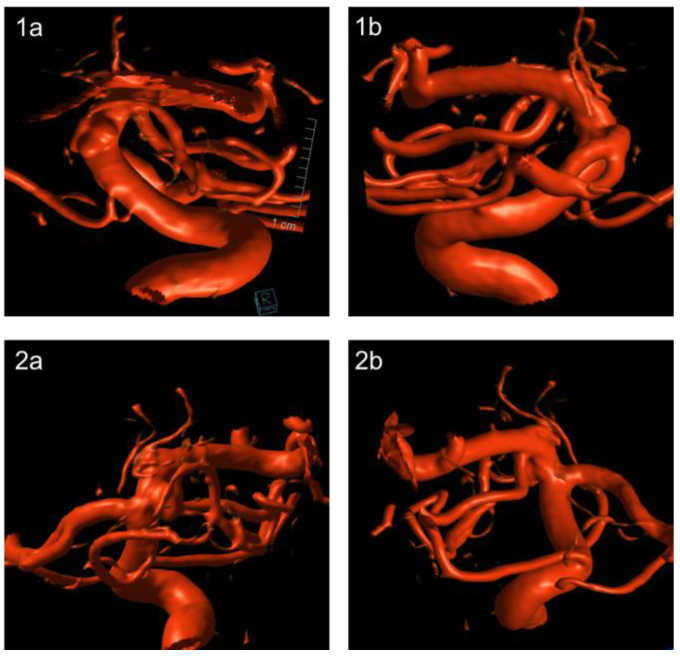
Presents a detailed depiction of the 3 mm aneurysm. Panel 1 illustrates a laterolateral projection, with (**1a**) viewed from the right and (**1b**) from the left. Panel 2 shows an anteroposterior projection, with (**2a**) from the posterior and (**2b**) from the anterior aspect.

**Figure 2:**
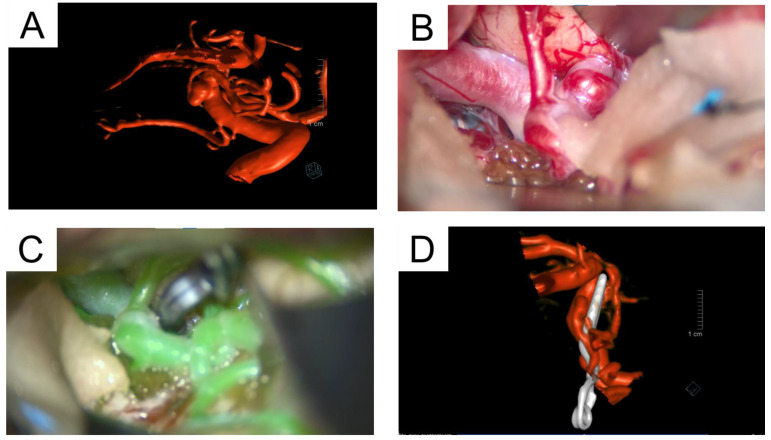
Representative images illustrating the diagnostic and surgical management of the proximal MCA aneurysm (**A**) Preoperative digital subtraction angiography (DSA) showing the unruptured proximal MCA aneurysm. (**B**) Intraoperative view of the exposed proximal MCA aneurysm before clipping. (**C**) Application of the aneurysm clip during the surgical procedure. (**D**) Postoperative DSA confirming complete exclusion of the aneurysm with preserved blood flow in adjacent vessels.

We report the case of a 46-year-old male who presented to the emergency department, following a transient ischemic attack. His symptoms included transient left-sided ptosis and needle-like paresthesia in the left arm, both of which resolved spontaneously. The patient was admitted to the stroke unit for treatment and observation. Brain magnetic resonance imaging (MRI) identified an incidental 3 mm broad-based aneurysm in the proximal M1 segment of the right MCA, oriented temporo-occipitally (Figure 1 and Figure 2A). The aneurysm was determined to be unrelated to his presenting symptoms.

At a follow-up visit in our outpatient clinic, the patient reported no ongoing neurological symptoms, including headaches, neck pain, or sensory disturbances. A comprehensive clinical examination was unremarkable, revealing no focal neurological deficits, no aphasia, and intact cranial nerve function. The patient demonstrated a stable gait with no evidence of meningeal irritation. Following a multidisciplinary discussion at the neurovascular board, the patient was informed that the aneurysm’s morphology and rupture risk warranted a relative indication for surgery. Upon explicit request, the elective surgical intervention was performed.

The aneurysm was accessed using a standard transsylvian approach (Figure 2B). Intraoperatively, a frontal basal branch originating directly from the aneurysm dome presented a significant challenge during clip application. Careful and precise clip placement, confirmed by intraoperative fluorescence angiography, achieved complete aneurysm occlusion while maintaining flow in the parent vessels (Figure 2C). Intraoperatively, pronounced vasospasm of the MCA was observed, requiring the administration of nimodipine to mitigate its effects.

**Figure 3:**
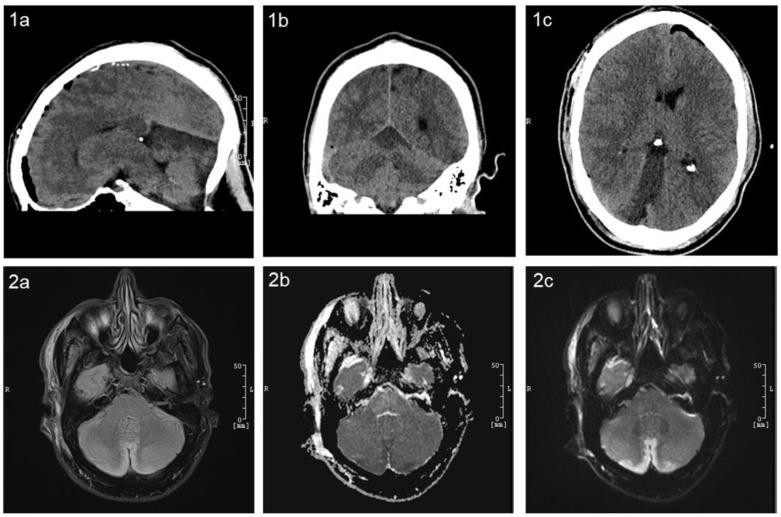
Non-contrast CT and MRI of the brain showing a hypodense area in the superior cerebellum, consistent with an infarction in the territory of the superior cerebellar artery (SCA). This finding correlates with the patient’s perioperative neurological symptoms. Panels (**1a**–**c**) show the postoperative CT scan in sagittal, coronal, and axial views. Panel (**2a**) displays the postoperative brain MRI in T2-FLAIR sequences, while panel (**2b**) presents diffusion-weighted imaging with apparent diffusion coefficient mapping sequences and (**2c**) present standard diffusion-weighted imaging sequences.

Postoperatively, anisocoria (right pupil larger than the left) was observed in the operating room, raising immediate concern. A prompt CT scan was performed, revealing no acute abnormalities and providing initial reassurance. Further investigation with CT perfusion and angiography showed no evidence of vasospasm or perfusion deficits. The clinical course took a significant turn a few hours later when the patient suffered a generalized tonic–clonic seizure, necessitating re-intubation in our neurosurgical ICU. Repeat imaging, conducted 3 hours after the initial CT, revealed a diffuse infarction, most likely located in the territory of the superior cerebellar artery (SCA), without clear territorial demarcation (Figure 3). The patient’s medical history and family reports suggested potential amphetamine use on the day before surgery, possibly even immediately before the procedure. A drug screening confirmed the presence of amphetamines, aligning with the family’s account. Amphetamines are known to remain detectable in urine for up to 3–4 days after consumption. Subsequent MRI imaging confirmed a cerebellar infarction bilaterally in the paramedial region, involving the vermis and the superior cerebellar artery’s supply area (Figure 3). The TOF sequence showed no evidence of occlusion in the major brain-supplying vessels.

Complementary stroke workup was conducted in parallel to imaging. A transthoracic echocardiogram and long-term ECG monitoring showed no signs of a cardiac embolic source or arrhythmia. No extracranial or intracranial stenosis was detected in the digital subtraction angiography already performed perioperatively, making atherosclerotic or embolic stroke mechanisms unlikely. After exclusion of other common etiologies and in light of the confirmed amphetamine use, a drug-induced vasospastic infarction was considered the most probable cause. Based on the results of the stroke workup, no indication for anticoagulation, antiplatelet therapy, or further specific stroke treatment was established. Anticoagulation was initiated later in the clinical course due to the development of deep vein thrombosis, but this treatment was not related to the management of the cerebellar infarction.

After successful weaning and extubation, the patient exhibited disorientation, likely due to withdrawal symptoms. Despite an otherwise uneventful hospital course, the patient chose to discharge himself against medical advice following a consultation with psychiatry colleagues. He did not attend further follow-up appointments.

## 3. Discussion

The occurrence of perioperative strokes is a rare but well-described complication in aneurysm surgery [11]. This phenomenon has been observed not only in the thoracic region [12] but also during the surgical clipping of cerebral aneurysms [13,14]. Non-specific factors, such as alterations in somatosensory evoked potentials (SSEPs) and the Hunt and Hess grading scale, have been previously evaluated for their association with the occurrence of perioperative strokes [14]. However, the Hunt and Hess grading scale is specifically designed for ruptured aneurysms and does not apply to incidental aneurysms treated electively, as in this case. Furthermore, intraoperative SSEP changes indicate real-time neurological events but cannot be used as a reliable predictive factor for perioperative strokes. The role of specific external factors predisposing patients to perioperative strokes remains insufficiently explored. Building on this, we present a potential connection between amphetamine abuse and the occurrence of a perioperative stroke in our patient undergoing elective aneurysm surgery.

During the surgery, a tendency toward vasospasms was observed in the MCA. Such vasospasms can occur following vascular manipulation, even in patients without a history of drug use. Irrigation with nimodipine successfully resolved the vasospastic changes, with no evidence of global vasospasm at any point. However, the undetected amphetamine use highlighted a critical gap in preoperative protocols, as it may have contributed to the cerebellar stroke via an amphetamine-induced vasospasm. The interplay between acute amphetamine use and neurosurgical intervention could create a precarious scenario with an elevated risk of perioperative complications [9,15]. This phenomenon has previously been documented in the coronary and splanchnic circulations [16], aligning with evidence that amphetamines exacerbate endothelial dysfunction and induce vasospasm, thereby potentially increasing the risk of ischemic events in high-risk surgical contexts [1]. A more thorough evaluation of the patient’s substance use history, coupled with routine toxicology screening, might have identified the amphetamine consumption prior to surgery. Such screening may have enabled postponement or modification of the planned intervention, potentially mitigating the risk of complications.

A further challenge arose in managing the patient’s disorientation during the prolonged weaning process, which was initially attributed to withdrawal symptoms. This attribution complicated the clinical assessment by obscuring the distinction between infarction-related neurological deficits and delirium. Amphetamine withdrawal is well documented to cause not only delirium but also psychosis [17,18,19], further complicating clinical evaluation.

In patients with chronic amphetamine use and stable vital signs as well as a normal ECG, anesthesia can generally be administered safely. However, caution is advised due to potential cardiovascular risks. β-blockers like propranolol may cause unopposed α-adrenergic stimulation and should be avoided. Blood pressure can be managed with agents such as nitroprusside, nitroglycerin, or dexmedetomidine. During acute intoxication, hemodynamic instability is common, presenting either as hypertension and hyperthermia or hypotension due to catecholamine depletion. In cases of ephedrine-resistant hypotension, phenylephrine may be effective. Ketamine and halothane should be avoided due to their potential for exacerbating cardiac complications [20]. Withdrawal symptoms such as anxiety, restlessness, and tremors can be managed with buspirone, ondansetron, and propranolol [21]. In patients with a history of heroin or methadone use, anesthesia and analgesia can be challenging, particularly in long-term users due to opioid-induced hyperalgesia. The variability in drug purity complicates equianalgesic opioid dosing. Continuation of opioids is necessary to prevent withdrawal, but a multimodal approach including acetaminophen, non-steroidal anti-inflammatory drugs, gabapentin, and pregabalin is recommended. Perioperative ketamine can help reduce opioid requirements and mitigate hyperalgesia [20].

Such scenarios emphasize the need for a multidisciplinary approach involving anesthesiology, neurology, and critical care teams to ensure a comprehensive assessment and tailored management strategy. Robust preoperative screening and vigilance in the postoperative period are essential to potentially mitigating similar outcomes in future cases.

This case report has a limitation due to its single-case nature and the absence of a control group, which restricts the ability to establish a definitive causal relationship between amphetamine use and perioperative stroke. As such, all conclusions drawn must be interpreted with caution and serve primarily as a basis for further investigation.

## 4. Conclusions

This case highlights the interplay between unrecognized amphetamine use and perioperative risk in intracranial aneurysm surgery. The acute amphetamine-induced vasospasm observed during the procedure, combined with the undetected substance use, may have contributed to a perioperative cerebellar stroke despite meticulous surgical and anesthetic management. This underscores the critical need for enhanced preoperative protocols, including routine toxicology screening, particularly in patients with a history of substance abuse, even when denied by the patient. Additionally, the complex postoperative presentation, initially attributed to withdrawal symptoms, emphasizes the importance of a multidisciplinary approach in distinguishing between infarction and delirium. Early identification of substance use and tailored intraoperative strategies, such as the prophylactic use of vasospasm-mitigating agents, are essential to potentially mitigate risks and improve outcomes in high-stakes neurovascular procedures. It also underscores the importance of a multidisciplinary approach involving anesthesiology, neurology, and critical care teams to ensure comprehensive assessment and individualized management strategies. Robust preoperative screening and heightened vigilance during the postoperative period are essential to reducing the risk of similar adverse outcomes in the future.

## Data Availability

No new data were created or analyzed in this study.

## Abbreviations

The following abbreviations are used in this manuscript:

MCA	middle cerebral artery
MRI	magnetic resonance imaging
SCA	superior cerebellar artery
SSEPs	somatosensory evoked potentials
